# Prognostic value of serological markers of hepatitis B virus infection in squamous cell cervical cancer

**DOI:** 10.7150/jca.61249

**Published:** 2021-09-13

**Authors:** Xingrao Wu, Lan Li, Yanqing Li, Meiping Jiang, Kangming Li, Zheng Li, Lan Zhang

**Affiliations:** 1 Department of Radiation Oncology, Yunnan Cancer Hospital, The Third Affiliated Hospital of Kunming Medical University, Yunnan Cancer Center, No. 519, Kunzhou Road, Kunming 650118, People's Republic of China.; 2 Department of Gynecologic Oncology, Yunnan Cancer Hospital, The Third Affiliated Hospital of Kunming Medical University, Yunnan Cancer Center, No. 519, Kunzhou Road, Kunming 650118, People's Republic of China.

**Keywords:** cervical cancer, hepatitis B virus, serologic markers, prognosis

## Abstract

**Objective:** The current study aimed to investigate the prognostic value of serological markers of hepatitis B virus (HBV) infection in squamous cell cervical cancer.

**Methods:** Squamous cell cervical cancer patients treated by concurrent chemoradiotherapy from January 2013 to December 2015 at Yunnan Cancer Hospital were retrospectively reviewed.

**Results:** Of a total of 277 patients, 12 (4.33%), 93 (33.57%), 2 (0.72%), 25 (9.02%), and 36 patients (13.00%) were seropositive for hepatitis B surface antigen (HBsAg), anti-hepatitis B surface antibodies (anti-HBs), hepatitis B envelope antigen (HBeAg), anti-hepatitis B envelope antibodies (anti-HBe), and anti-hepatitis B core antibodies (anti-HBc), respectively. No patients experienced more than mild hepatic adverse events during treatment. The five-year overall survival (OS) rates for patients with anti-HBs positive or negative status were 85.8% and 66.2% (*p* = 0.039), respectively. No statistically significant difference in the five-year OS rates was observed in HBsAg positive and negative, HBeAg positive and negative, anti-HBe positive and negative, anti-HBc positive and negative patients. The multivariable analysis revealed that anti-HBs positivity was an independent favorable prognostic factor for OS (HR= 0.279; 95%CI: 0.083-0.936;* p* = 0.039) in patients younger than 50 years.

**Conclusions:** The presence of anti-HBs predicts a superior OS for squamous cell cervical cancer patients aged younger than 50 years.

## Introduction

Cervical cancer is a leading cause of cancer-related morbidity and mortality among women in developing countries. Despite advances in diagnostic and screening techniques and a wider availability of vaccines, a significant proportion of patients are diagnosed with local advanced disease at the initial visit 1. For the majority of patients who present with squamous cell carcinoma, concurrent chemoradiotherapy is the primary treatment modality for local advanced disease 2. The International Federation of Gynecology and Obstetrics (FIGO) staging system is a reliable method for determining clinical treatment strategies and predicting treatment outcome 2; however, additional prognostic factors are required to improve outcome prediction and tailor individualized treatment for patients with cervical cancer 3, 4.

HBV infection represents the most comment chronic viral infection in the world. Approximately 30% of the world's population exhibits serological evidence of a current or previous HBV infection 5. In addition, HBV infection is associated with an increased risk of both hepatocellular carcinoma and extra-hepatic malignancies 6-11. Moreover, HBV infection has been found to be associated with the prognosis of malignancies. Current or past HBV infection was shown to be an adverse prognostic factor in patients with nasopharyngeal carcinoma 12, lung cancer 13, breast cancer 14, ovarian cancer 15, diffuse large B cell lymphoma 16-18, and pancreatic cancer 19. The latent mechanism leading to the unfavorable prognosis in HBV-infected patients with malignancies may be linked to certain types of immunological dysfunction 20, HBV reaction 21 or the presence of Hepatitis B X-interacting protein, which plays an oncogenic role in cancer progression 22. Given that HBV can be transmitted by sexual contact, it is plausible that HBV may interact with human papillomavirus to promote the development and progression of cervical cancer. However, whether and to what extent HBV infection impacts clinicopathological characteristics and prognosis of cervical cancer remains to be determined.

The great majority of previous studies considered hepatitis B surface antigen (HBsAg) as marker of HBV infection. Less attention has been paid to the role of other HBV-related antibodies and antigens, including antibody to hepatitis B surface antigen (anti-HBs), a protective body in HBV infection, hepatitis B e antigen (HBeAg), hepatitis B envelope antibody (anti-HBe), antibody to hepatitis B core antigen (anti-HBc), the marker of past exposure to HBV on patient survival. In this study, we aimed to evaluate the different baseline serological markers of HBV infection with cervical cancer survival in a region where both HBV and cervical cancer are endemic.

## Methods

### Ethical considerations

This retrospective study was approved by the Research Ethical Committees of the Third Affiliated Hospital of Kunming Medical University (Kunming, China). Informed consent was waived by the committee due to the retrospective nature of this study. The confidentiality of the patient status was maintained by avoiding personal identifiers during analysis.

### Patient Selection

Squamous cell cervical cancer patients who received radical radiotherapy were consecutively recruited from January 2013 to December 2015. All patients were selected according to the clinical information extracted from the Electronic Medical Record (EMR) system. The inclusion criteria were as follows: (1) confirmed histological diagnosis of cervical squamous cell cancer; (2) completion of concurrent chemoradiotherapy with definitive-intent; (3) serological markers of HBV infection, including HBsAg, anti-HBs, HBeAg, anti-HBe, anti-HBc before treatment; (4) ≥ 20 and ≤ 70 years old; (5) Easter Cooperative Oncology Group (ECOG) ≤ 1. Patients were excluded if they met the following criteria: (1) had previously received any anticancer therapy; (2) coinfection with hepatitis C virus (HCV) and/or human immunodeficiency virus (HIV), or with an unknown HCV or HIV status; (3) a history of previous or synchronous malignant tumors; (4) pregnant of lactating; and (5) unsuitable for chemotherapy due to a liver, kidney, lung, or heart deficiency. Diagnoses of cervical squamous cell cancer were based on the comprehensive cervical cancer control guidelines of the World Health Organization (WHO). Cancers were staged by two experienced gynecologic oncologists based on a vaginal examination, chest and abdomen computed tomography (CT) with iodine-containing contrast medium, and pelvic magnetic resonance imaging (MRI), or positron emission tomography-CT (PET-CT) according to the FIGO 2009 standard. Lymph nodes with a short-axis diameter over 10 mm or relative apparent diffusion coefficient (ADC) values in imaging studies, or image findings of ring enhancement and marked necrosis, were defined as lymphadenopathy.

### Treatment

All patients were treated with definitive-intent concurrent chemoradiotherapy. Most patients (130 of 277; 46.9%) were treated with 2-dimensional conventional radiotherapy (2-DRT), whereas 42.6% (118 of 277) received intensity-modulated radiotherapy (IMRT), and the remaining 10.5% (29 of 277) received 3-dimensional conformal radiotherapy (3-DCRT). Patients received 45 Gy - 50.4 Gy external beam radiotherapy delivered to the pelvis in 23-28 fractions of 1.8 Gy - 2.0 Gy, with concurrent weekly cisplatin/nedaplatin chemotherapy at a dose of 25 mg/m^2^ - 40 mg/m^2^ for four to six cycles, and high dose rate intracavity brachytherapy of 20 Gy - 30 Gy in 4-5 fractions beginning at week five of external radiotherapy.

### Virological measurements and liver function studies

Patients were screened for serological HBsAg, anti-HBs, HBeAg, anti-HBe, and anti-HBc using enzyme-linked immunosorbent assay (WANTAI BioPharm, Beijing, China) at the time of diagnosis at Yunnan Cancer Hospital. Patients were also tested for the presence of antibodies specific for serum HIV, hepatitis A virus, HCV, hepatitis D virus, and hepatitis E virus using enzyme-linked immunosorbent assay (WANTAI BioPharm, Beijing, China). The cut-off values for the different hepatitis B markers were set according to their average values of the negative control samples provided by the manufacturer of each commercial kit. The quality control for the measurement of the hepatitis B markers was performed in accordance with the manufacturer's protocols. Briefly, the test results for all positive and negative quality control samples were required to be correctly classified as indicated in each kit. Inconsistent results for quality control samples prompted repeated testing. In addition, a routine external quality assessment with the pooled serum provided by the Ministry of Health of the People's Republic of China was conducted daily. Specific workers were responsible for sending blood samples for tests in time to guarantee the quality of the test. In most cases, the tests were only performed as a qualitative detection. HBV deoxyribonucleic acid (HBV-DNA) was not routinely detected.

All patients were monitored using routine liver function tests, including analyses of AST, ALT, and the total bilirubin levels every week during treatment. The severity of hepatic dysfunction and adverse events were defined according to the National Cancer Institute Common Toxicity Criteria (version 4.0).

### Follow-up

After primary treatment, the patients were advised to receive regular follow-ups every three months for the first two years, every six months for the following three years, and annually thereafter. Patients who did not visit our hospital as scheduled were telephoned for follow-up to obtain the patient's status. The final follow-up occurred in January 2019. In the event of patient death, families were questioned about the cause of death. Overall survival (OS) was the primary endpoint that was evaluated, which was calculated from the first day of diagnosis to death or to the date of the last follow-up.

### Statistical analysis

SPSS software (version 17.0, SPSS Inc., Chicago, IL) was used for all statistical analyses. Categorical variables in each of the different groups were compared using a chi-squared test or a Fisher's exact test, where indicated. Actuarial rates were calculated using the Kaplan-Meier method, and differences were compared using a log-rank test. A Cox regression was used for the univariate and multivariate analyses. Hazard ratios (HR) with their 95% confidence intervals (CI) were computed using the Cox proportional-hazards model. Covariates, including host factors (i.e., age and hemoglobin), tumor factors (i.e., tumor size, differentiation, and lymph node status), and radiotherapy techniques were included in all tests. The threshold for statistical significance was set an α of 0.05 and all *p* values were based on two-sided tests.

## Results

### Baseline Patient Characteristics

A total of 277 cases qualified for the analysis. All of the patients were from Southwestern China and were primarily distributed in the provinces of Yunnan and Guizhou. Of the 277 total patients, there were 12 patients (4.33%) who were seropositive for HBsAg, 93 patients (33.57%) seropositive for anti-HBs, 2 patients (0.72%) seropositive for HBeAg, 25 patients (9.02%) seropositive for anti-HBe, and 36 patients (13%) seropositive for anti-HBc. Table 1 summarizes the characteristics of these patients and their tumors. The HBsAg positive and negative groups were similar regarding host factors, histological categories, and tumor factors, as were the anti-HBs positive and negative groups, HBeAg positive and negative groups, anti-HBe positive and negative groups, and anti-HBc positive and negative groups (Table 2). There were no significant differences regarding the use of radiotherapy techniques between these groups (Table 2).

### Hepatic Adverse Events

According to the liver enzyme and total bilirubin levels, no patients experienced moderate, severe, or life-threatening hepatic adverse events during concurrent chemoradiotherapy. However, we were unable to reliably ascertain hepatic adverse events that occurred after treatment completion since several patients received subsequent follow-up care at local facilities.

### Follow-up of Study Participants

The median follow-up period for the 277-patient cohort was 44 months, with a maximum follow-up duration of 72 months. It is important to note that two types of follow-up were employed in this study. One group of patients underwent physical and/or imaging examinations for recurrent disease as scheduled. In contrast, the other group of patients did not return for examination. The survival of patients who did not return for examination were confirmed through telephone contact, and the precise onset of disease recurrence is unknown. Overall, as of the last day of follow-up, 204 patients (73.65%) were alive with tumor-free or stable disease, 60 patients (21.66%) died, and 13 patients (4.69%) were lost to follow-up. The death of one patient (1.67%) was attributed to a traffic accident; however, the patient was also diagnosed with cancer locoregional recurrence and distant metastasis. All of the other deaths were attributed to cancer (98.33%).

### Prognostic value of serological markers of HBV infection in cervical cancer patients

**Figure 1:**
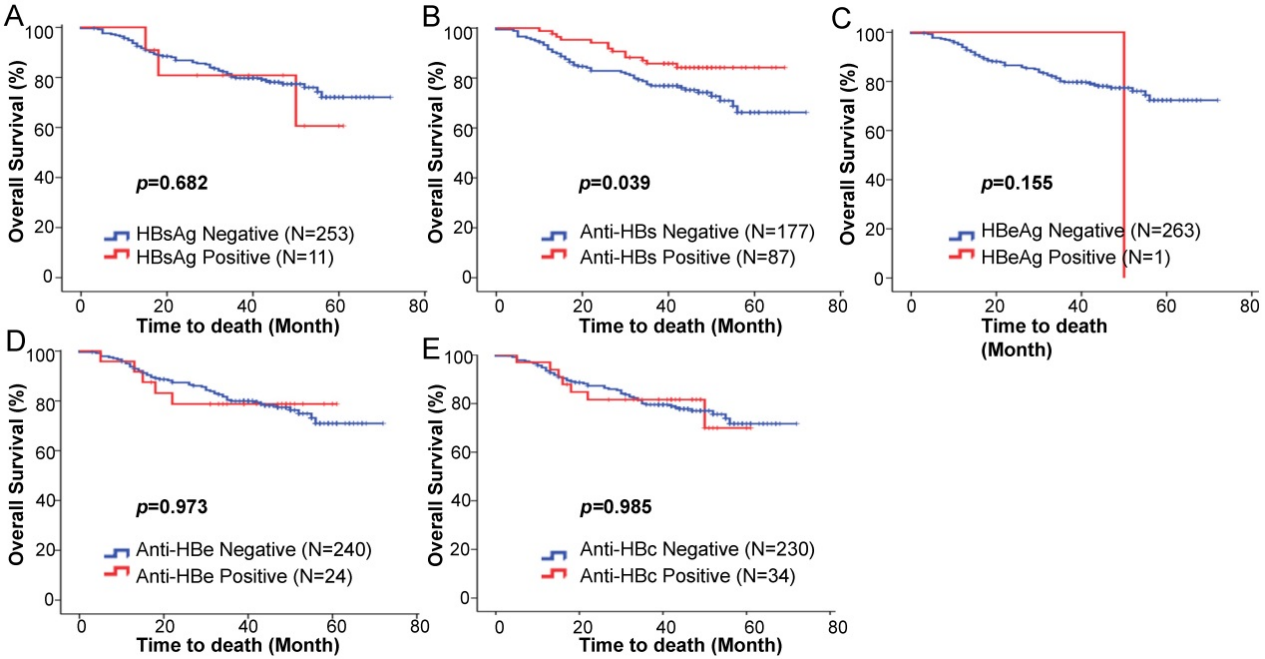
Kaplan-Meier estimated overall survival based on the (**A**) HBsAg, (**B**) anti-HBs, (**C**) HBeAg, (**D**) anti-HBe, (**E**) anti-HBc status among squamous cell cervical cancer patients treated with concurrent chemoradiotherapy.

We hypothesized that there may be a prognostic value of the baseline serological HBV markers on survival outcome. The Kaplan-Meier analysis and log-rank test showed no statistically significant differences in the five-year OS rates between HBsAg positive and negative patients (76.0% vs 72.1%; *p* = 0.682) (Fig. 1A). The five-year OS rate of the anti-HBs positive patients was significantly higher than that of the anti-HBs negative patients (85.8% vs 66.2%; *p* = 0.039) (Fig. 1B). The baseline HBeAg status was not associated with changes in the five-year OS (*p* = 0.155; Fig. 1C). No statistically significant differences were observed for the five-year OS rates between the anti-HBe positive and negative groups (78.8% vs 70.9%; *p* = 0.973) (Fig. 1D), and the anti-HBc positive and negative groups (69.9% vs 71.7%; *p* = 0.985) (Fig. 1E).

In the univariate analysis, patients with cervical cancer who were anti-HBs positive had significantly superior OS compared with the anti-HBs negative patients (HR = 0.530; 95%CI, 0.287-0.980;* p* = 0.043) (Table 3). In the multivariate analysis, after adjusting for risk factors, including clinical stage, tumor size, histological differentiation, lymph node status, and hemoglobin, anti-HBs was not found to be an independent prognostic factor for OS (HR = 0.788; 95%CI: 0.578-1.074;* p* = 0.132) (Table 3). The upper limit of the 95% CI nearly reached 1, indicating that anti-HBs positivity may be a potential favorable prognostic factor for OS.

**Figure 2:**
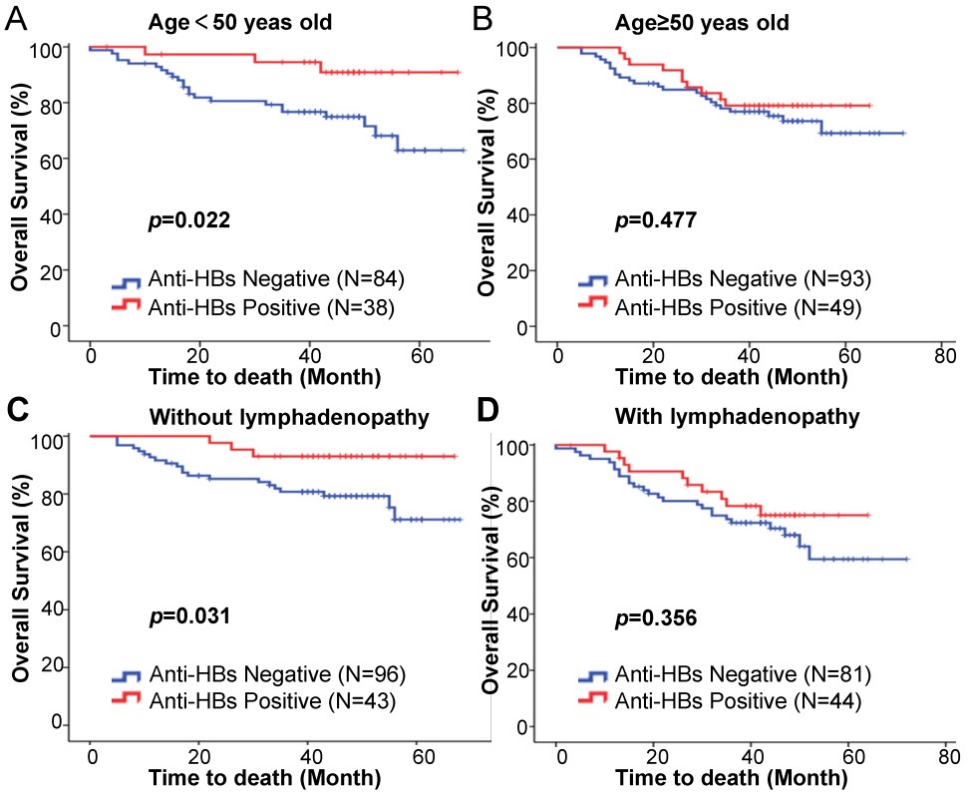
Kaplan-Meier estimation of overall survival based on the anti-HB status among patients (**A**) aged < 50 years, (**B**) aged ≥ 50 years, (**C**) without lymphadenopathy, and (**D**) with lymphadenopathy.

We further examined the prognostic value of anti-HBs in the different subgroups stratified according to age, clinical stage, tumor size, tumor differentiation, lymph node status, and hemoglobin. The effect of HBsAg differed significantly by patient age or lymph node status, with a favorable effect for patients aged younger than 50 years old (HR = 0.269; 95%CI: 0.081-0.896;* p* = 0.039; Table 4) and without lymphadenopathy (HR = 0.287; 95%CI: 0.086-0.962;* p* = 0.043; Table 4). The five-year OS was significantly higher in the anti-HBs positive patients in the under 50 years subgroup (90.9% vs 74.9%; *p* = 0.022; Fig. 2A) and without lymphadenopathy subgroup (93.0% vs 79.3%; *p* = 0.031; Fig. 2C). This was not the case for the ≥ 50 years old subgroup (HR = 0.766; 95%CI: 0.366-1.603; *p* = 0.480; Table 4) (79.2% vs 73.6%; *p* = 0.477; Fig. 2B), with lymphadenopathy subgroup (HR = 0.711; 95%CI: 0.342-1.475;* p* = 0.359; Table 4) (75.0% vs 68.0%; *p* = 0.356; Fig. 2D). The effect of anti-HBs on patient survival was not significant in the subgroups stratified by clinical stage (Supplementary Fig. 1A), tumor size (Supplementary Fig. 1B), tumor differentiation (Supplementary Fig. 1C), and hemoglobin (Supplementary Fig. 1D) (Fig. 2). Among the patients aged younger than 50 years old, anti-HBs positivity was found to be an independent prognostic factor for superior OS (HR = 0.279; 95%CI: 0.083-0.936;* p* = 0.039) (Table 5).

## Discussion

Although a vaccination subsidy was provided to cover all infants and subsequently led to a substantial reduction in the HBV infection rate among Chinese children, HBV infection remains highly prevalent in populations over 5 years of age 23. HBV infection is associated with dysfunction. The association between HBV infection and poor prognosis is keeping demonstrated in a variety of cancers, including nasopharyngeal carcinoma 12, lung cancer 13, breast cancer 14, ovarian cancer 15, diffuse large B cell lymphoma 16-18, and pancreatic cancer patients 19. We speculated HBV infection may have an impact on the prognosis of cervical cancer patients.

HBV is a hepatotrophic and lymphotrophic virus that can infect and replicate in lymphocytes and peripheral blood mononuclear cells 24. HBsAg is a serum marker of an ongoing HBV infection, including chronic hepatitis B and the inactive HBsAg carrier state. Theoretically, primary HBV infections spontaneously resolve in 95% of adults. However, in less than 10% of cases, the virus is contracted during the perinatal period, which can lead to persistent viral replication and chronic HBV infection (cases considered to be HBsAg positive). Anti-HBc is a serum marker for a current or previous HBV infection. Low levels of HBV replication in the serum or liver are considered to indicate a resolved HBV infection (HBsAg negative and anti-HBc positive) 25. An HBV infection may increase susceptibility to chronic inflammation, DNA damage, and cancer development 26.

HBsAg or anti-HBc positivity have been demonstrated to be risk factors for extrahepatic cancers, such as gastric cancer 10, nasopharyngeal carcinoma 9, pancreatic cancer 26, biliary tract cancer 11, colorectal cancer, kidney cancer, ovarian cancer, and non-Hodgkin's lymphoma 12. A few previous studies have assessed associations between HBV infection and cervical cancer. However, results of those studies were consistent. In a study by An et al. 27, cervical cancer was significantly related to HBV infection with a relative risk of ~1.49. Wei et al. 28 indicated cervical cancer might be HBV-related (adjusted odds ratio = 1.22; 95%CI: 1.05-1.42). The study of Siu et al. 29 found patients with malignant or pre-malignant cervical lesion have increased risk of becoming hepatitis B carrier. By contrast, nonsignificant association were observed between HBV infection and cervical cancer in other studies 6-8.The prevalence of HBsAg and anti-HBc positive patients with cervical cancer in this study were 4.3% (12 of 277) and 13.0% (36 of 277), respectively, lower than that of the general population in China (7.2% and 34.1%, respectively) 30. Due to HBsAg positive patients with elevated baseline liver enzymes who did not complete concurrent chemotherapy were not included, the prevalence of HBsAg positive patients may be underestimated in this study. Whether HBV infection plays a role in cervical cancer development requires future population-based prospective studies or qualified case-control studies.

HBsAg positive patients with cancer, including nasopharyngeal carcinoma 12, lung cancer 13, breast cancer 14, ovarian cancer 15, diffuse large B cell lymphoma 16-18, and pancreatic cancer 19 have an inferior OS. Previous studies speculated that it may be linked to certain types of immunological dysfunction 20 HBV reactivation 21, or the presence of Hepatitis B X-interacting protein 22. Given that HBV can be transmitted by sexual contact, it is plausible that HBV may be present in the epithelium of the cervix and promote the progression of cervical cancer. Subsequent systemic chronic inflammation and the altered cytokine network following HBV infection might also be relevant in cervical cancer progression. However, there were no significant association between HBsAg status and OS in cervical cancer patients in the present study. Interestingly, the anti-HBs positive cervical cancer patients had a significantly superior OS compared with the anti-HBs negative patients. Anti-HBs is a protective antibody in HBV infection. As suggested by the clinical value of anti-HBs, anti-HBs positivity may result from both an HBV infection or hepatitis B vaccination. We considered that the positive effect of anti-HBs may actually reflect the negative effect of HBV infection. Occult HBV infection has been described in patients negative for HBsAg who have been previously exposed to HBV and recovered from acute or chronic infection 31, 32. After HBsAg disappearing, the systemic inflammation and HBV DNA may still persist in the body of the previous infection individuals and forced the body to react abnormally 25, 33. Using HBsAg positive as an HBV infection indicator may omit the occult infection. Anti-HBs, which indicates the immunity of HBV, may potentially be of more clinical importance to be a predictor of patient survival than HBsAg.

However, why the prognostic impact of anti-HBs was not statistically significant for the total patients but was significant for patients younger than 50 years old remains unclear. It is reported the median ages at diagnosis and death of HBsAg positive cancer patients were significantly younger than those with HBsAg negative 28. Li et al. 14 reported HBV infection was an unfavorable prognostic predictor in very young patients. Le et al. 34 reported that HBsAg levels declined with age in HBV infected patients. The oncogenic role of HBV infection seems to be age-dependent. The younger the infection, the more obvious the impact in patient cancer progression. The positive effect of anti-HBs in patients younger than 50 years old may be results of the negative effect of HBV infection. Future studies are warranted to elucidate the legitimacy and detailed mechanisms associated with the role of anti-HBs in cervical cancer.

Our study nevertheless has certain limitations. First, this was a retrospective study and has weak efficacy for testing prognostic factors that have a direct causal relationship. Secondly, the information regarding tumor control and relapse was incomplete, which restricted the analysis to disease free survival. Thirdly, due to a lack of data regarding the HBV DNA load, the survival of patients who exhibited a high HBV replication rate remains unknown. However, despite these limitations, our findings provide a rationale for further studies.

## Conclusion

In conclusion, this study is the first to evaluate the prognostic value of serological markers of HBV infection in cervical cancer patients in China, where both diseases are endemic. Anti-HBs positivity was found to be an independent favorable prognostic factor in squamous cell cervical cancer patients aged younger than 50 years old and treated with concurrent chemoradiotherapy. Further prospective studies involving large cohorts of patients with cervical cancer are warranted to confirm these results. In addition, the latent mechanism by which anti-HBs is associated with a superior survival outcome in cervical cancer patients requires further investigation.

## Supplementary Material

Supplementary figure.

## Figures and Tables

**Table 1:** Clinical characteristics

Characteristics	All Patients (N=277)	No. (%)
**Age, years**		
<50	128	46.2
≥50	149	53.8
**FIGO stage**		
I-II	195	70.4
III-IV	82	29.6
**Tumor size (cm)**		
<4	68	24.5
≥4	209	75.5
**Histological differentiation**		
Well and moderate	182	65.7
Poor	70	25.3
Papillary squamous carcinoma	25	9.0
**Lymphadenopathy**		
No	151	54.5
Yes	126	45.5
**Hemoglobin (g/L)**		
<120	84	30.3
≥120	193	69.7
**Radiotherapy techniques**		
2-DRT	130	46.9
3-DCRT	29	10.5
IMRT	118	42.6
**HBsAg**		
Negative	265	95.7
Positive	12	4.3
**Anti-HBs**		
Negative	184	66.4
Positive	93	33.6
**HBeAg**		
Negative	275	99.3
Positive	2	0.7
**Anti-HBe**		
Negative	252	91.0
Positive	25	9.0
**Anti-HBc**		
Negative	241	87.0
Positive	36	13.0

Abbreviations: HBsAg, hepatitis B surface antigen; anti-HBs, hepatitis B surface antibody; HBeAg, hepatitis B envelope antigen; anti-HBe, hepatitis B envelope antibody; anti-HBc, hepatitis B core antibody; 2-DRT, 2-dimensional radiotherapy; 3-DRT,3-dimensional radiotherapy; IMRT, intensity-modulated radiotherapy; FIGO, International Federation of Gynecology and Obstetrics.

**Table 2:** Relationships between HBV serological markers and patient characteristics

Characteristics	HBsAg	HBsAg	*p*	Anti-HBs	Anti-HBs	*p*	HBeAg	HBeAg	*p*	Anti-HBe	Anti-HBe	*p*	Anti-HBc	Anti-HBc	*p*
	(-)	(+)		(-)	(+)		(-)	(+)		(-)	(+)		(-)	(+)	
**Age, years**			0.124			0.524			0.213			0.837			0.474
<50	120	8		88	40		126	2		117	11		109	19	
≥50	145	4		96	53		149	0		135	14		132	17	
**FIGO stage**			0.772			0.481			0.505			0.854			0.893
I-II	187	8		127	68		194	1		177	18		170	25	
III-IV	78	4		57	25		81	1		75	7		71	11	
**Tumor size (cm)**			0.182			0.126			0.418			0.674			0.728
<4	67	1		40	28		68	0		61	7		60	8	
≥4	198	11		144	65		207	2		191	18		181	28	
**Histological differentiation**			0.764			0.186			0.591			0.204			
Well and moderate	173	9		118	64		180	2		169	13		159	23	0.598
Poor	68	2		52	18		70	0		60	10		59	11	
Papillary squamous carcinoma	24	1		14	11		25	0		23	2		23	2	
**Lymphadenopathy**			0.786			0.491			1			0.876			0.394
No	144	7		103	48		150	1		137	14		129	22	
Yes	121	5		81	45		125	1		115	11		112	14	
**Hemoglobin (g/L)**			0.817			0.086			0.544			0.239			0.674
<120	80	4		62	22		83	1		79	5		72	12	
≥120	185	8		122	71		192	1		173	20		169	24	
**Radiotherapy techniques**			0.765			0.539			0.887			0.819			0.742
2-DRT	125	5		82	48		129	1		119	11		113	17	
3-DCRT	27	2		20	9		29	0		27	2		24	5	
IMRT	113	5		82	36		117	1		106	12		104	14	

Abbreviations:(+), positive; (-), negative; HBsAg, hepatitis B surface antigen; anti-HBs, hepatitis B surface antibody; HBeAg, hepatitis B envelope antigen; anti-HBe, hepatitis B envelope antibody; anti-HBc, hepatitis B core antibody; 2-DRT, 2-dimensional radiotherapy; 3-DRT,3-dimensional radiotherapy; IMRT, intensity-modulated radiotherapy; FIGO, International Federation of Gynecology and Obstetrics. *p* value was calculated using the chi-square test or Fisher exact test if indicated.

**Table 3:** Univariate and multivariate Cox hazards analysis between clinical features and OS

Characteristics	Univariate analysis		Multivariate analysis	
	HR (95%CI)	*p*-value	HR (95%CI)	*p*-value
**Age, years**				
≥50	1 (Reference)			
<50	1.113 (0.668-1.854)	0.682		
**FIGO stage**				
I-II	1 (Reference)		1 (Reference)	
III-IV	2.100 (1.258-3.504)	**0.006**	1.665 (0.968-2.863)	0.065
**Tumor size (cm)**				
<4	1 (Reference)		1 (Reference)	
≥4	1.989 (0.979-4.039)	0.057	1.176 (0.815-1.698)	0.386
**Histological differentiation**				
Well and moderate	1 (Reference)		1(Reference)	
Papillary squamous carcinoma	0.425 (0.102-1.766)	0.239		
Poor	1.883 (1.107-3.204)	**0.019**	1.322 (0.881-1.982)	0.177
**Lymphadenopathy**				
No	1 (Reference)			
Yes	1.873 (1.106-3.115)	**0.019**	1.797 (1.071-3.017)	**0.027**
**Hemoglobin (g/L)**				
≥120	1 (Reference)			
<120	3.227 (1.942-5.361)	**<0.001**	1.678 (1.282-2.198)	**<0.001**
**RT techniques**				
2-DRT	1 (Reference)			
3-DCRT	0.925 (0.357-2.398)	0.872		
IMRT	1.100 (0.647-1.868)	0.725		
**Anti-HBs**				
Negative	1 (Reference)		1 (Reference)	
Positive	0.530 (0.287-0.980)	**0.043**	0.788 (0.578-1.074)	0.132

Abbreviations: 95%CI, 95% confidence interval; FIGO, International Federation of Gynecology and Obstetrics; 2-DRT, 2-dimensional radiotherapy; 3-DRT,3-dimensional radiotherapy; IMRT, intensity-modulated radiotherapy; anti-HBs, hepatitis B surface antibody; HR, hazard ratio. *p* value was calculated using a Cox proportional hazards model.

**Table 4:** Subgroup analysis by anti-HBs status for OS in squamous cell cervical cancer patients

Prognostic factor	OS (months)	Effect estimate	
	Mean	HR (95%CI)	*p*-value
***Age (years)***			
**<50**			**0.032**
Anti-HBs negative	53.7	1 (Reference)	
Anti-HBs positive	63.5	0.269 (0.081-0.896)	
**≥50**			0.48
Anti-HBs negative	58.2	1 (Reference)	
Anti-HBs positive	56.5	0.766 (0.366-1.603)	
***Lymphadenopathy***			
**No**			**0.043**
Anti-HBs negative	56.8	1 (Reference)	
Anti-HBs positive	64.1	0.287 (0.086-0.962)	
**Yes**			0.359
Anti-HBs negative	54.2	1 (Reference)	
Anti-HBs positive	54.4	0.711 (0.342-1.475)	
***FIGO stage***			
**I-II**			0.145
Anti-HBs negative	56.9	1 (Reference)	
Anti-HBs positive	61.9	0.554 (0.251-1.225)	
**III-IV**			0.235
Anti-HBs negative	50.3	1 (Reference)	
Anti-HBs positive	53.6	0.554 (0.208-1.470)	
***Tumor size (cm)***			
**<4**			0.66
Anti-HBs negative	63	1 (Reference)	
Anti-HBs positive	60.8	0.732 (0.183-2.931)	
**≥4**			0.061
Anti-HBs negative	53.1	1 (Reference)	
Anti-HBs positive	59.5	0.516 (0.258-1.030)	
***Hemoglobin (g/L)***			
**<120**			0.276
Anti-HBs negative	45.2	1 (Reference)	
Anti-HBs positive	51.8	0.611 (0.251-1.485)	
**≥120**			0.198
Anti-HBs negative	62.3	1 (Reference)	
Anti-HBs positive	62.3	0.570 (0.242-1.341)	
***Histological differentiation***			
**Well and moderate**			0.283
Anti-HBs negative	59.7	1 (Reference)	
Anti-HBs positive	60.5	0.671 (0.323-1.391)	
**Poor**			0.39
Anti-HBs negative	48.2	1 (Reference)	
Anti-HBs positive	44.8	0.584 (0.172-1.987)	
**Papillary squamous carcinoma**			0.507
Anti-HBs negative	40.5	1 (Reference)	
Anti-HBs positive	48.3	0.020 (0.000-2089.644)	

Abbreviations: 95%CI, 95% confidence interval; FIGO, International Federation of Gynecology and Obstetrics; anti-HBs, hepatitis B surface antibody; HR, hazard ratio. *p* value was calculated using a Cox proportional hazards model.

**Table 5:** Multivariate analysis of prognostic factors for patients <50 years old

Characteristics	Multivariate analysis	
	HR (95%CI)	*p*-value
**FIGO stage**		**0.015**
≥50	1 (Reference)	
<50	2.808 (1.220-6.461)	
**Tumor size (cm)**		0.664
I-II	1 (Reference)	
III-IV	1.135 (0.641-2.012)	
**Histological differentiation**		0.146
Well and moderate	1 (Reference)	
Poor	1.559 (0.857-2.834)	
**Lymphadenopathy**		**0.034**
No	1 (Reference)	
Yes	1.558 (1.004-2.342)	
**Hemoglobin (g/L)**		**0.007**
≥120	1 (Reference)	
<120	3.342 (1.388-8.046)	
**Anti-HBs**		**0.039**
Negative	1 (Reference)	
Positive	0.279 (0.083-0.936)	

Abbreviations: 95%CI, 95% confidence interval; FIGO, International Federation of Gynecology and Obstetrics; anti-HBs, hepatitis B surface antibody; HR, hazard ratio. *p* value was calculated using a Cox proportional hazards model.

## Abbreviations

OS: Overall survival
2-DRT: 2-dimensional radiotherapy
3-DRT: 3-dimensional radiotherapy
HBsAg: hepatitis B surface antigen
anti-HBs: hepatitis B surface antibody
HBeAg: hepatitis B envelope antigen
anti-HBe: hepatitis B envelope antibody
anti-HBc: hepatitis B core antibody
IMRT: intensity-modulated radiotherapy
FIGO: International Federation of Gynecology and Obstetrics
ECOG: Easter Cooperative Oncology Group
HCV: hepatitis C virus
HIV: human immunodeficiency virus
WHO: World Health Organization
MRI: magnetic resonance imaging
PET-CT: positron emission tomography-computed tomography
ADC: apparent diffusion coefficient
DNA: deoxyribonucleic acid
HR: Hazard ratios
CI: confidence intervals
PD-1: programmed cell death protein
AST: aspartate aminotransferase
ALT: alanine aminotransferase
ULN: upper limit of normal
