# Supplementary material for: Prognostic value of serological markers of hepatitis B virus infection in squamous cell cervical cancer
Source: J Cancer. 2021 Sep 13;12(22):6620–8. doi: 10.7150/jca.61249 (PMC8518014; doi:10.7150/jca.61249)
Supplement: Supplementary file 1 — Supplementary figure. [file jcav12p6620s1.pdf]

Supplementary Figure 1

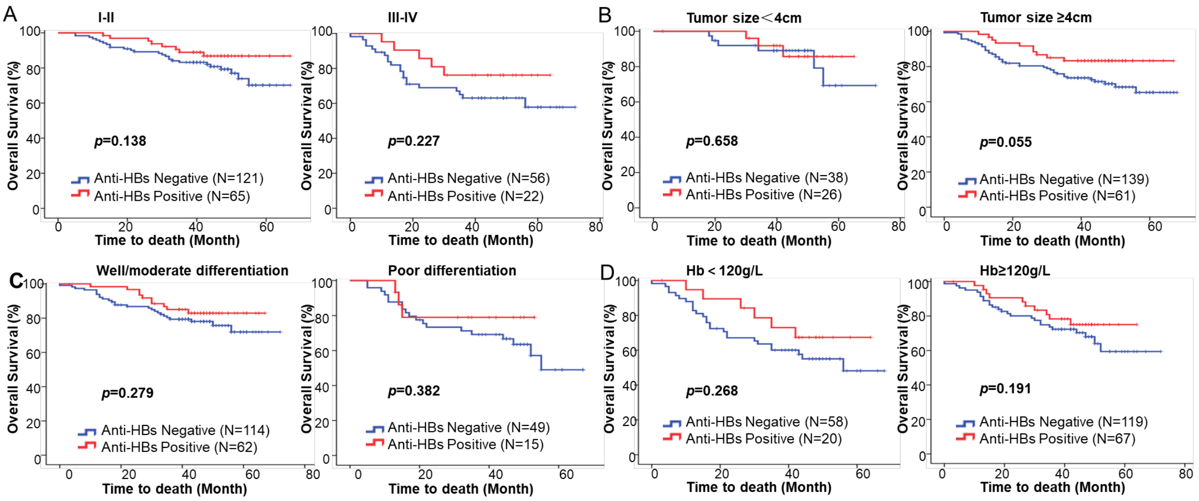

Kaplan-Meier estimated overall survival by anti-HBs status among patients (**A**) with FIGO stage I-II (left) or III-IV (right), (**B**) with tumor size ＜4cm (left) or ≥4cm, (**C**) with well/moderate differentiation (left) or poor differentiation (right), (**D**) with Hemoglobin＜120g/L (left) or ≥120g/L (right).
